# Technology-Assisted Behavioral Intervention to Extend Sleep Duration: Development and Design of the Sleep Bunny Mobile App

**DOI:** 10.2196/mental.8634

**Published:** 2018-01-10

**Authors:** Kelly Glazer Baron, Jennifer Duffecy, Kathryn Reid, Mark Begale, Lauren Caccamo

**Affiliations:** ^1^ Rush University Medical Center Chicago, IL United States; ^2^ Department of Psychiatry University of Illinois at Chicago Chicago, IL United States; ^3^ Center for Circadian and Sleep Medicine Feinberg School of Medicine Northwestern University Chicago, IL United States; ^4^ Department of Preventive Medicine Feinberg School of Medicine, Northwestern University Chicago, IL United States

**Keywords:** sleep duration, wearable, obesity, technology, behavioral intervention

## Abstract

**Background:**

Despite the high prevalence of short sleep duration (29.2% of adults sleep <6 hours on weekdays), there are no existing theory-based behavioral interventions to extend sleep duration. The popularity of wearable sleep trackers provides an opportunity to engage users in interventions.

**Objective:**

The objective of this study was to outline the theoretical foundation and iterative process of designing the “Sleep Bunny,” a technology-assisted sleep extension intervention including a mobile phone app, wearable sleep tracker, and brief telephone coaching. We conducted a two-step process in the development of this intervention, which was as follows: (1) user testing of the app and (2) a field trial that was completed by 2 participants with short sleep duration and a cardiovascular disease risk factor linked to short sleep duration (body mass index [BMI] >25).

**Methods:**

All participants had habitual sleep duration <6.5 hours verified by 7 days of actigraphy. A total of 6 individuals completed initial user testing in the development phase, and 2 participants completed field testing. Participants in the user testing and field testing responded to open-ended surveys about the design and utility of the app. Participants in the field testing completed the Epworth Sleepiness Scale and also wore an actigraph for a 1-week baseline period and during the 4-week intervention period.

**Results:**

The feedback suggests that users enjoyed the wearable sleep tracker and found the app visually pleasing, but they suggested improvements to the notification and reminder features of the app. The 2 participants who completed the field test demonstrated significant improvements in sleep duration and daytime sleepiness.

**Conclusions:**

Further testing is needed to determine effects of this intervention in populations at risk for the mental and physical consequences of sleep loss.

## Introduction

According to data from the National Health Interview Survey, 70.1 million adults (29.2%) sleep <6 hours per a 24-hour period [1]. Short sleep duration is thought to contribute to the development of chronic illnesses, including hypertension, overweight, and diabetes, as well as increased risk for all-cause mortality [2-4]. A recent consensus panel determined that the evidence suggests that *at least 7 hours of sleep* is the recommended sleep duration for adults [5]. Increasing awareness of short sleep duration as one of the US health priorities is noted in Healthy People 2020 [6].

A growing number of studies have demonstrated potential benefits of increasing sleep duration among individuals with short sleep duration. Studies conducted among small samples have demonstrated reductions in beat-by-beat blood pressure among individuals with hypertension and prehypertension [7], improvements in insulin resistance among prediabetics [8], reductions in appetite among overweight individuals [9], improvements in athletic performance among athletes [10], and improvement in some areas of cognitive functioning among adolescents [11,12]. These studies have focused on sleep extension under tightly controlled conditions for short periods of time. Typically, individuals with short sleep duration (<6 or <7 hours) were asked to increase time in bed by going to bed earlier and, if applicable, waking up later. Participants were tracked with sleep diaries, wrist actigraphy, and contact with study staff.

Building upon this research, our aim is to develop scalable behavioral interventions for reducing short sleep duration with the aim of improving health and well-being. The determinants of short sleep duration are multifactorial, with some but not all aspects amenable to intervention. For example, work schedules and caregiving responsibilities are often not modifiable. Epidemiologic research has demonstrated that short sleep duration is more prevalent in males than in females, more prevalent among blacks compared with whites, and more prevalent in middle-aged adults than in older adults [13-16]. Time use surveys indicate that sleep time is often exchanged for work and commute time [17]. A handful of studies have evaluated social/cognitive theories of sleep behavior. Attitudes, social norms, and perceived control were all areas that predicted behavioral intention to sleep 7 to 8 hours per night in college students [18]. In another study that used the health belief model, self-efficacy was the strongest predictor of sleep duration. Finally, Kroese and colleagues utilized principles of self-control theory to coin the term “bedtime procrastination” and reported associations between bedtime behavior with self-control and sleep duration [19]. These studies suggest that psychological and behavioral targets for interventions may be successful at extending sleep duration using behavioral interventions that target motivation and self-efficacy.

The rapidly expanding prevalence of wearable sleep-tracking devices provides the opportunity to engage a large number of individuals in sleep behavior change. It is estimated that 1 in 10 adults owns a wearable fitness device [20]. As of 2015, it is estimated that 33 million adults in the United States own a fitness device, many of which have sleep-tracking capabilities. However, there are currently no validated technology-assisted interventions to extend sleep duration. Existing technology interventions for sleep have been designed for insomnia [21,22] or treatment adherence in obstructive sleep apnea (eg, MyAir by ResMed Inc). Given the prevalence of short sleep duration and the popularity of sleep tracking, we designed a technology-assisted behavioral intervention to extend sleep duration: the “Sleep Bunny.” The goal of this paper was to describe the theoretical foundation, development, and present two case examples from field testing the Sleep Bunny intervention, a technology-assisted sleep extension intervention.

## Methods

### Participants

The general inclusion criteria for all participants included age between 18 and 65 years and self-reported sleep duration <7 hours per night. Exclusion criteria included the following: unstable or serious medical conditions (eg, neurological conditions, cancer); shift work; diagnosis of obstructive sleep apnea or high risk for apnea based on screening questionnaires; symptoms of other sleep disorders, including insomnia and restless legs syndrome; current series of unstable psychiatric disorders such as schizophrenia, bipolar, alcohol abuse, or drug abuse; and pregnancy or desire to become pregnant in the study period. We had separate recruitment strategies for each study phase, which were as follows: (1) user testing and (2) field testing.

For user testing, we recruited healthy volunteers from a list of participants who completed screening for a previous study [23] but were ineligible for the study because of sleep duration <7 hours. They were primarily medical students who were willing to evaluate our app, provide feedback, and identify potential problems in the tools. For the field test, we recruited participants with body mass index (BMI) >25, to test our fully functioning intervention in a population with at least one health risk factor linked to short sleep duration (overweight/obesity). The reason that participants with BMI>25 were selected was to have a specific health issue to focus the coaching sessions around (eg, links between sleep and weight). Participants for the field test were recruited from the community using online advertisements and flyers posted on campus. This study was approved by the Northwestern University Institutional Review Board, and enrolled participants provided written informed consent.

### Procedure

#### User Testing

This phase of intervention development was used to elicit initial feedback from participants and identify and address any problems with the app. Eligible participants from our previous study were invited for a preintervention visit to the laboratory, which included questionnaires, setup and training of the Fitbit and mobile phone app, and actigraphy. Participants started with 1 baseline week at home where they entered sleep diaries but were asked not to alter their habitual sleep schedule. At the end of week 1, participants had the initial telephone coaching session (20 min) to review goals and motivation and troubleshoot any problems with the app or Fitbit. Participants had 4 weekly coaching sessions (10 min or less), with sessions 3 and 4 conducted via text or email if preferred. At the end of the 4-week intervention, participants returned to the laboratory to complete questionnaires and interviews about their experience. During this phase, participants received the coaching sessions as scheduled, but content from the intervention was in most cases delivered differently due to resolving initial problems in the app. For example, 3 participants were not able to view their Fitbit data in the app, and 4 participants’ mobile phone app did not advance the didactic modules each week as planned. When possible, the study staff worked around these issues (eg, delivering content via email rather than the app, had participants view their Fitbit data in the Fitbit app if the Sleep Bunny app was not pulling in the data) to deliver the relevant intervention components. All issues with the technology were addressed before moving on to the next phase of the study (field testing).

#### Field Testing

We recruited participants from the community to complete a small field test to determine the feasibility and acceptability of the fully functioning intervention. The field test was conducted using the same procedure we described for user testing but was performed only after the app was fully functioning. Participants completed a pretreatment training session/questionnaires, 1 week of baseline monitoring, 4 weeks of intervention including weekly telephone or email/text coaching sessions, and a final follow-up visit at the end of the intervention to complete questionnaires and exit interviews. Participants received intervention components as intended, including content delivery and Fitbit integration.

### Intervention Description

The Sleep Bunny program is a technology-assisted behavioral intervention aimed at increasing sleep duration by extending time in bed. The developers named the program the “Sleep Bunny” because it agrees with the graphics theme of sleeping animals and also follows the app-naming convention of nonsense- or silly-sounding words for technology products (eg, Grooveshark, Foursquare, Hulu). The intervention content and coaching techniques were based on elements of motivational interviewing, and cognitive behavioral therapy was based on a telephone coaching manual for an online depression intervention [24]. Participants in this intervention were provided with a mobile phone app and wearable sleep tracker (Fitbit), and they then participated in weekly brief telephone coaching sessions. Coaches were able to view participants’ intervention engagement and data through a dashboard. The goal of the coaches’ dashboard was to increase supportive accountability [25], a concept in technology-assisted behavioral interventions that suggests individuals are more likely to change their behavior if they are accountable to another person.

The app included the following components: (1) content, which is delivered in weekly “lessons,” (2) interactive tools, (3) sleep tracking, (4) graphic feedback, and (5) reminders/notifications.

#### Intervention Content

**Figure 1:**
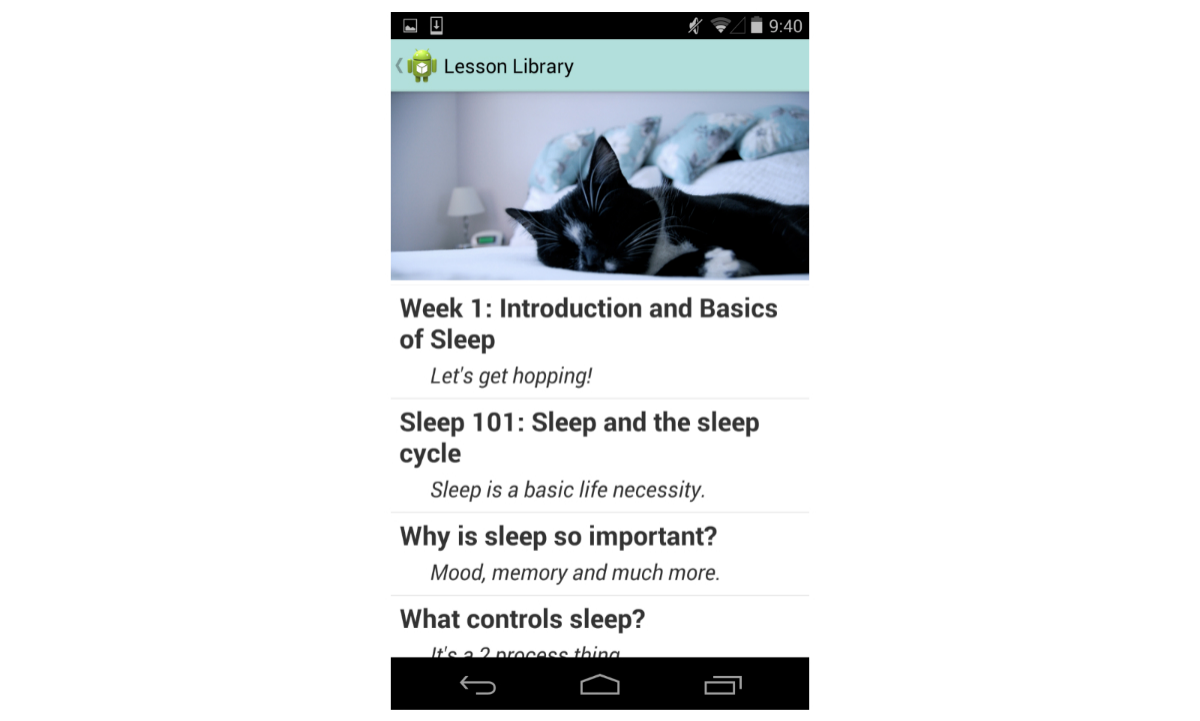
Lesson, week 1, from the mobile phone app.

An example of intervention content is listed in Figure 1. Participants received 4 weekly lessons, which included written didactic content via the mobile app. Lesson topics included the basics of sleep, beating bedtime procrastination, circadian rhythms, and relapse prevention. A new lesson was released each week, as new content can draw users back to an intervention. Participants also had access to past lessons.

#### Intervention Tools

Participants had access to a bedtime checklist, where they could organize and track activities they planned to complete before their scheduled bedtime; a daily sleep diary, which could be viewed on the dashboard by the telephone coach; and a “bedtime procrastination quiz” [19], where they could compare their scores with published values. Coaches could view all data entered in tools and were expected to discuss with participants.

#### Sleep Tracking

Participants wore a Fitbit Flex sleep-tracking device for the 4-week intervention. The in-house development core at the Northwestern University Center for Behavioral Intervention Technology used the Fitbit open application programming interface to pull in the participant’s data into the mobile app. Coaches were also able to see the participant’s sleep tracker data on the coach’s dashboard. Coaches were able to log in to a dashboard to view sleep diary, app use (eg, frequency of lessons and tools used), and Fitbit data including the bedtime, wake-up time, sleep duration, and number of awakenings per night.

#### Graphic Feedback

**Figure 2:**
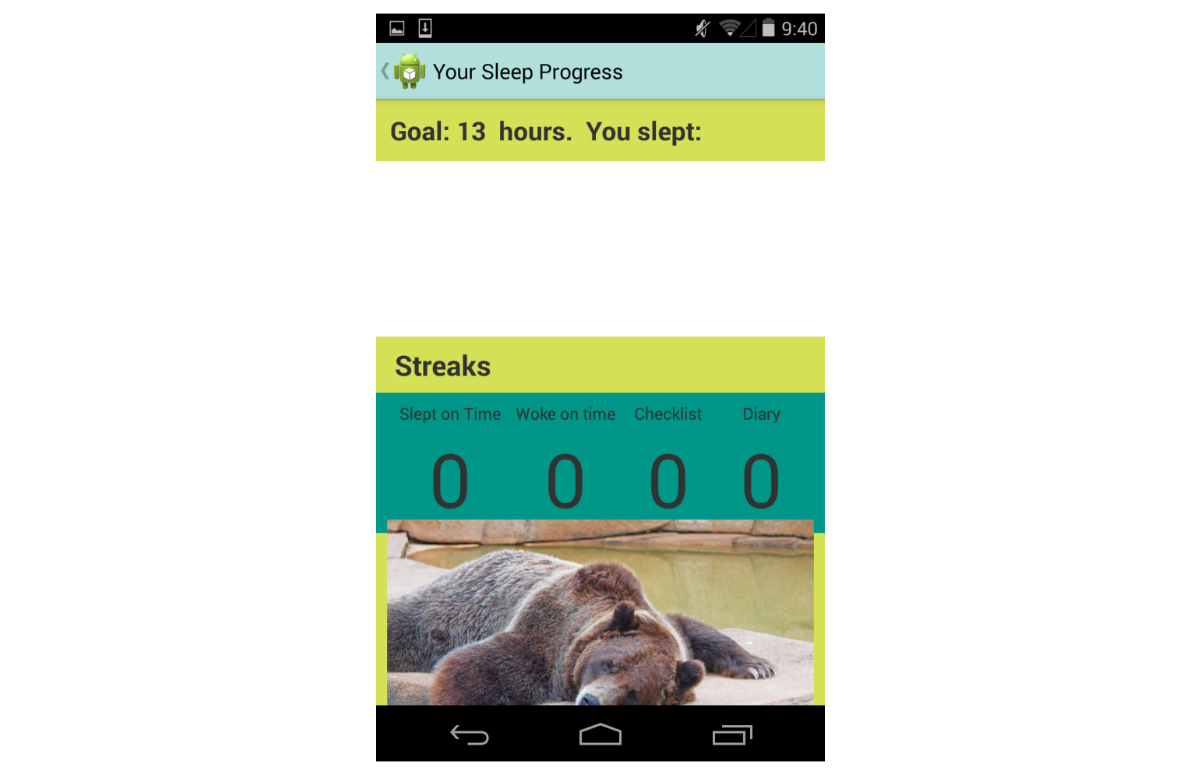
Feedback and streaks from the mobile phone app.

A “graphs” section of the app displayed individuals’ daily sleep duration (Figure 2). Individuals were also given feedback about “streaks” of days in a row when they achieved their goals of completing a sleep diary, target bedtime, wake-up time, and sleep duration.

#### Reminders and Notifications

Participants received a prompt to complete sleep diaries if the diary was not completed within 4 hours of their scheduled wake-up time via a notification that was presented on the home screen. The app also included an alarm clock, tied into the wake-up time goals set in the app. Participants could clear the notification from their home screen, similar to a text message. The alarms could be silenced but could only be changed by changing the goals in the app.

#### Coaching

Participants were assigned to a sleep coach to monitor their progress during the study (KB or LC) and were provided weekly telephone coaching sessions related to their sleep-related goals. The coaching protocol, developed by Drs Duffecy and Baron, is based on the principles of supportive accountability and based on a previous coaching protocol [24,25]. The coaches worked with participants to establish sleep-related goals with the participants based on the participant’s values and beliefs, including the particular sleep schedule goals and also usage goals (eg, number of Fitbit wear days or sleep logs per week or goal bedtime/wake-up time). Coaches asked participants to enter their bedtime and wake-up time goals into the app, which would ensure the delivery of reminders and alarms at the appropriate time. Coaches monitored participants’ intervention usage through an online dashboard. The first coaching session was a 20-min engagement session, which included introductions, rational for the program, clarifying roles of the coach, and the participants’ goals for the program, such as their target bedtime and wake-up time. For weeks 2-4, participants had weekly brief (10 min) follow-up support calls to review their weekly Fitbit data and troubleshoot any problems with the app or Fitbit as well as answer any questions about the weekly content. Weeks 3 and 4 coaching sessions could be completed over email or text if the participant preferred these modes of communication. In between sessions, the coaches were available, mostly over email, to troubleshoot any problems with the app or Fitbit.

### Measures

#### Sleep Disorders Screening

Before enrollment into the study, participants completed the STOP questionnaire to be screened for sleep apnea risk [26]. Participants scoring as high risk on this measure were excluded.

#### Psychiatric Disorders Screening

Participants completed self-reported questions on the presence of psychiatric conditions and medications and completed the patient health questionnaire 8-item (PHQ-8) version. Participants were excluded if they reported the presence of serious or unstable psychiatric disorders (bipolar or schizophrenia) or dementia or scored >10 on the PHQ-8.

#### Sleep

Participants wore an Actiwatch [27] Spectrum (Philips/Respironics, Inc, Bend, Oregon) on the nondominant wrist during the 4-week intervention to provide a validated measure of sleep duration. Due to poor specificity for detecting sleep among consumer sleep-tracking devices, actigraphy was considered the sleep outcome assessment despite participants wearing a Fitbit to deliver the intervention. Actiwatches were set with 30-second epoch length and medium sensitivity. Actigraphic sleep parameters were calculated using Actiware-Sleep 6.0 software with default settings and included the following variables: sleep onset time, sleep offset time, sleep duration, wake after sleep onset (WASO), and sleep efficiency (sleep duration/time in bed). Off-wrist time was excluded based on the Spectrum’s off-wrist detection. Actigraphy profiles were scored using procedures previously reported [28]. Participants were trained on the use of the event marker. When the event marker or sleep diary was not available, research staff used decrease in light and activity as a guide to delineate the rest period.

Daytime sleepiness was measured using the Epworth Sleepiness Scale (ESS), an 8-item self-report measure [29]. Scores on the ESS range from 0 to 24, and scores ≥10 are considered to be excessive sleepiness [30].

Participants responded to open-ended questions on a user experience survey at the end of user testing and intervention. The survey asked participants to provide comments on the content, layout, graphics, and general feedback about the app. Responses were analyzed by thematic analysis.

## Results

### User Testing

We conducted user testing with 6 individuals. Participants were primarily female (4 women, 2 men) and in mid-20s (average age 24.6 years, standard deviation [SD] 4.4 years). Of the participants who attended the first study visit, 2 individuals were excluded or dropped out (1 participant was excluded due to overnight work not disclosed at screening, and 1 participant withdrew without reason). An additional participant arrived for the baseline visit but did not provide consent because she had changed her sleep duration since the time she had answered the screening questions. In total, 4 participants completed the 4-week intervention. During this testing period, we discovered and fixed problems in the app (eg, lessons not advancing each week, problems with the app not integrating with Fitbit data) and tested the coaching procedures. Approximately half of the participants used their own android phones and the other half borrowed study phones (Wi-Fi only, no cellular plan).

### Field Testing

We recruited 3 participants for the small field test and 2 completed the 4-week intervention and assessments. The participant who did not complete the 4-week intervention and assessments because of technical issues (unable to load the app on her phone or the study phone) was withdrawn from the intervention. All the 3 participants who volunteered for the target population user testing were female: 1 participant was black, 1 was white, and 1 was Asian. Average age was 51 (SD 2.1) years. All were working full time. Daytime sleepiness ratings on the ESS ranged from 3 to 16, with an average of 9.7 and SD of 5.3. Of the 3 volunteers, 2 had ESS scores ≥10.

### App Feedback (From User Testing and Field Testing)

A summary of user feedback is presented in Textbox 1. In general, participants reported that the format was pleasant and the lessons were understandable. All participants reported enjoying use of the wearable sleep tracker. The greatest amount of constructive feedback was focused on improving the notifications. Participants reported they were either missed or inappropriate (eg, did not know how to turn off the alarm on weekends).

### Field Test Results: Adherence and Response to Intervention in Two Cases

The participants in the field test completed 100% of coaching sessions and 50% to 60% of sleep diary days and wore Fitbit 50% to 80% of nights. Table 1 reports changes in actigraphy and subjective sleepiness. The 2 participants who completed the field test demonstrated a large increase in sleep duration and a large decrease in ESS score. Changes in WASO and sleep efficiency were in opposite directions for the 2 participants: 1 participant demonstrated small improvements in WASO and sleep efficiency, and the other participant had a moderate increase in WASO (+23 min) but a small change in sleep efficiency (−4%).

**Responses to open-ended questions during user testing and field testing.**

Appearance

- “Pleasant”
- “Animals were cute”
- “I liked the little dog”
- “I would like videos”

Lessons

- “Too long”
- “It was basic but a good refresher and reinforced in the coaching”
- Terminology
- “Bedtime procrastination, I didn’t know what that was”
- “I don’t look at it as procrastination because I have so many things to do”

Notifications

- “The evening prompt was nice”
- “I wanted to turn it off [the morning alarm] on the weekends but didn’t know how”
- “I never saw the notifications but was not using my phone [was a study phone]”

Ease of use

- “I didn’t know how to change the goals”
- “The goals were easy to adjust on the phone but hard in life”
- “The diary was tedious to record”

Duration of intervention

- “4 weeks is a reasonable time”
- “3-4 weeks”
- “I think it would take 2 months to figure out how to change my life’’

Other comments

- “This was a stressful time and the intervention changed my life. I didn’t meet my goal but I would have been sleeping so much less if I wasn’t in the study”

**Table 1:** Actigraphy and sleepiness scores.

Participant	Baseline sleep duration (hours)	Week 4 sleep duration (hours)	Baseline sleep efficiency (%)	Week 4 sleep efficiency (%)	Baseline WASO^a^ (min)	Week 4 WASO (min)	Baseline ESS^b^	Week 4 ESS
1	5.75	7.35	87	90	42	32	7	0
2	4.30	6.0	85	81	25	48	18	9

^a^Wake after sleep onset.

^b^Epworth Sleepiness Scale.

## Discussion

### Principal Findings

This study outlines the theoretical foundation and development process of a novel technology-assisted behavioral intervention to extend sleep duration. Results indicate that we have developed an app that is visually pleasing to participants and content is appropriate. Coaching sessions were well attended and not burdensome to participants. Participants enjoyed the use of the wearable sleep tracker most of all. We pilot tested the intervention in a small field test of participants and reported a case series of 2 participants. These participants demonstrated a substantial improvement in sleep duration and a reduction in self-reported daytime sleepiness, but changes to WASO and sleep efficiency were not consistent.

The improvements in sleep duration in our 2 participants were larger than those previously reported in several sleep extension interventions [7,8], and on par with the findings of Tasali and colleagues [9]. The magnitude of change may be high due to the low baseline sleep duration in our target population participants, which allowed for greater change in sleep and the use of weekly brief telephone coaching. The reason we selected participants with BMI>25 was to ensure that there was a health issue linked to sleep that we could discuss in the coaching, to enhance motivation. The addition of technology alone does not always improve adherence and outcomes. A recent weight loss trial demonstrated lower weight loss at 2-year follow-up among participants randomized to wear activity trackers [31]. Future research is needed to determine which components of sleep extension are most effective as well as cost-effective.

Our study also demonstrated variability in response to the intervention, even within the 2 cases studied. Although sleep duration increased in both participants, 1 participant had an increase in WASO after 4 weeks of the intervention and the other participant had a decrease. In this case, both participants improved in their daytime sleepiness, but there may be some individuals who respond poorly to sleep extension. For example, among patients with insomnia, restricting time in bed rather than extending it is the recommended treatment [32].

It is also important to note that studies comparing consumer sleep-tracking technology have found that these devices are far less accurate than actigraphy or polysomnography [27]. Given that consumer devices overestimate sleep duration due to being less sensitive to brief awakenings in the night, it is possible that they may provide false security about sleep duration to consumers. On the other hand, our experience in this development and field testing was that participants felt the measures reflected their sleep behaviors and the feedback was helpful with being far less intrusive than polysomnography. It may be that even flawed information is better than no information, because of the increase in awareness and self-monitoring. For example, it is well known that food diaries significantly underestimate caloric intake [33] but are the cornerstone of effective weight loss interventions [34]. Furthermore, although actigraphy is widely accepted as a valid estimation of sleep/wake-up patterns and sleep duration in naturalistic settings, actigraphy itself is less sensitive to awakenings and thus overestimates sleep duration compared with polysomnography [28].

### Strengths and Limitations

Limitations to our intervention include that our app was compatible with android only, and therefore excluded about half of all mobile phone users (iPhone), and our participants were highly educated, which may limit generalizability to other populations, particularly those with less familiarity with technology and mobile phone apps and may have a different response to the tracking and reminders in the app. Participant feedback was clear that they felt the notifications could be more targeted (eg, missed hearing bedtime reminder) or less annoying (did not know how to turn off the alarm on weekends, when they would have preferred to sleep later than on weekdays). Finally, although these results support the initial feasibility of our intervention, they provide only preliminary feedback and results of cases who participated in our early testing.

### Conclusions

In conclusion, this study outlines the theoretical foundation and development and presents a case series from early user testing of a technology-assisted sleep extension intervention. Future research is needed to continue to develop and refine this intervention and also explore maintenance of sleep behavior change.

## Abbreviations

BMI: body mass index
ESS: Epworth Sleepiness Scale
PHQ-8: patient health questionnaire 8-item
WASO: wake after sleep onset
